# Neurotrophic Activity of Cultured Cell Line U87 is Up-Regulated by Proline-Rich Polypeptide Complex and Its Constituent Nonapeptide

**DOI:** 10.1007/s10571-015-0192-8

**Published:** 2015-04-05

**Authors:** Agnieszka Zabłocka, Małgorzata Mitkiewicz, Józefa Macała, Maria Janusz

**Affiliations:** Department of Immunochemistry, Ludwik Hirszfeld Institute of Immunology and Experimental Therapy, Polish Academy of Sciences, Rudolfa Weigla 12, 53-114 Wrocław, Poland; Department of Microbiology, Ludwik Hirszfeld Institute of Immunology and Experimental Therapy, Polish Academy of Sciences, Rudolfa Weigla 12, 53-114 Wrocław, Poland

**Keywords:** Proline-rich polypeptide complex (PRP), Nonapeptide (NP), Human astrocytoma cell line U87, Nerve growth factor (NGF), Brain-derived neurotrophic factor (BDNF), Interleukin-6, Neuroprotection

## Abstract

Neurotrophins such as nerve growth factor (NGF) and brain-derived neurotrophic factor, as well as cytokines, for example, interleukin-6 (IL-6) play an important role in neuroprotection and in the control of the central nervous system (CNS) function. Reduced expression of neurotrophic factors can lead to dysregulation of neuron function and neuronal death. There is also evidence for mutual interactions between neurotrophins and IL-6. Therefore, the up-regulating the level of neuroprotective substances is one of the key manners to control the nervous system development and function. It can be a promising aim in the therapy of neurodegenerative disease in which the decreased level of neurotrophins is observed. In our recent studies, the role of proline-rich polypeptide complex (PRP) and its nonapeptide fragment (NP) in the regulation of neurotrophic activity in cultured astrocytes was shown. PRP and NP stimulate human astrocytoma cell line U87 to release the significant amounts of NGF to the extracellular space both in its precursor and mature form. We also provide the evidence that in NP-treated cells, the level of βNGF mRNA was increased. NP-treated cells used in this study produced also increasing amounts of IL-6. This finding indicates that PRP and its nonapeptide fragment NP up-regulate neurotrophic activity of U87 cell line by increase of NGF synthesis and its release into the extracellular space. It was also shown that NP-dependent increased production of IL-6 can enhance the NGF activity.

## Introduction

The proper development and function of the central nervous system (CNS) fundamentally depends on interaction between astrocytes and neurons. Astrocytes maintain homeostasis in undamaged CNS and provide structural, metabolic, and trophic support to neurons (Schipke and Kettenmann 2004; Perea et al. 2014). They can produce and release a wide spectrum of factors promoting neurogenesis, controlling survival and differentiation of neural cells, and modulating the CNS immune system (Markiewicz and Lukomska 2006; Sofroniew and Vinters 2010; Pirttinaki and Parri 2013). Under pathological conditions astrocytes are the main source of neurotrophin released in the CNS in response to harmful stimulants (Villoslada and Genain 2004; Farina et al. 2007; Angelowa and Abramow 2014).

Growth factors—nerve growth factor (NGF) and brain-derived neurotrophic factor (BDNF)—play a crucial role in intracellular communication, neuronal growth, and differentiation and are necessary for the survival and function of specific population of neural cells (Lessmann et al. 2003; Scaper 2008; Allen et al. 2013). NGF is responsible for maintaining neuronal phenotype (especially cholinergic) in the adult CNS including the regulation of the steady-state number of synapses (Liberto et al. 2004; Markiewicz and Lukomska 2006; Twiss et al. 2006; Scaper 2008). NGF is synthesized as a precursor, pro-NGF, which is cleaved to the mature form (Bruno and Cuello 2006). It is not clear whether the pro-NGF is converted to its mature form intracellularly or extracellularly. It was reported that brain cells are able to release pro-NGF into the extracellular space where the enzymatic conversion of pro-NGF to mature NGF form takes place (Lee et al. 2001; Bruno and Cuello 2006).

In the past decade, a growing interest in the participation of cytokines, especially interleukin-6 (IL-6) in neuroprotection was noticed. In the CNS Il-6 is synthesized mainly by microglia and astrocytes (Gruol and Nelson 1997; Perigolo-Vicente et al. 2013) but its role in the brain is still very controversial (for references see Barkho et al. 2006). Nevertheless, the role of IL-6 in the pathogenesis of neurodegenerative diseases as well as its physiological role in the CNS is under consideration (Gruol and Nelson 1997). There are increasing evidences supporting a key role of IL-6 in neurogenesis, neuronal growth, survival, differentiation and regeneration (Sterneck et al. 1996; März et al. 1999; Otten et al. 2000; Balschun et al. 2004; Barkho et al. 2006; Oh et al. 2010; Perigolo-Vicente et al. 2013). In addition, the role of IL-6 in the control of memory and learning processes was also investigated (Balschun et al. 2004).

During the last few years, many authors have demonstrated some evidence indicating the mutual interactions between neurotrophins and cytokines. It was shown that IL-6 may play a role as an enhancer of NGF signaling in neurons (Sterneck et al. 1996; Otten et al. 2000; Oh et al. 2010). The cultured sympathetic and sensory neurons, retinal ganglion cells as well as PC12 cells require IL-6 for survival, morphological/biochemical differentiation, and regeneration (März et al. 1999; Balschun et al. 2004; Oh et al. 2010; Perigolo-Vicente et al. 2013). The literature data indicate the relation of IL-6 with BDNF expression in neurons (Bartkowska et al., 2010).

A large body of evidences indicates that reduced expression of neurotrophins and changes in the level of their receptors can lead to dysregulation of neuronal function, and neuronal death can lead to the development of neurodegenerative diseases such as Alzheimer’s disease and Parkinson’s disease (Scaper 2008; Bruno et al. 2009; Calissano et al. 2010). Therefore, the possibility to up-regulate the level of neuroprotective substances, such as NGF, BDNF, and IL-6 is one of the key aspects to improve nervous system development and function, and can be a promising goal in the therapy of neurodegenerative diseases, in which a decreased level of neurotrophins is observed (Lessmann et al. 2003; Aloe et al. 2012; Allen et al. 2013). Because of difficulties with the use of recombinant neurotrophins in the therapy, increasing attention has been turned to alternative strategies. One promising possibility seems to be the use of bioavailable, naturally occurring substances with regulatory activities. Such a candidate can be the proline-rich polypeptide (PRP) complex obtained from ovine colostrum and its nonapeptide fragment (NP).

The PRP complex isolated from ovine colostrum shows multidirectional activity affecting the immune and nervous systems and seems to restore the balance in cellular function. The results obtained up to now indicate that the PRP complex possesses immunoregulatory properties, including effects on adaptive and innate immune responses, modulation of the functional/phenotypic differentiation of cells (Janusz et al. 1981; Kruzel et al. 2001; Janusz and Zabłocka 2010), shows prosurvival and proneuritogenic activities (Basci et al. 2005; Douraghi-Zadeh et al. 2009; Zabłocka et al. 2014), and has the ability to prevent the formation of amyloid β fibrils as well as to dissolve the aggregates already formed (Schuster et al. 2005; Bourhim et al. 2007; Janusz et al. 2009). Activities similar to the whole PRP complex were reflected by its nonapeptide fragment VESYVPLFP (NP) (for references see Janusz and Zabłocka 2010). Properties of PRP, its role in the development of the immune system, and cognitive function suggested its potential use in the treatment of neurodegenerative disorders including Alzheimer’s disease (Bilikiewicz and Gauss 2004).

In the present study, the effect of PRP and its constituent peptide (NP) on the up-regulation of neurotrophic activity was investigated in human astrocytoma cell line U87 which is used as an alternative model to study the cellular mechanisms in neuroprotection (Deb et al. 1999; Satpute et al. 2006; Wang et al. 2010a; Li et al. 2014). The effect of the peptides on the synthesis and release of neurotrophic substances such as NGF and interleukin-6 was studied.

## Materials and Methods

### Materials

High-glucose Dulbecco’s modified Eagle’s medium (DMEM), phosphate-buffered saline (pH 7.4) (PBS), and trypsin solution were from the Laboratory of General Chemistry of the Institute of Immunology and Experimental Therapy, PAN (Wrocław, Poland). Tissue culture dishes and fetal bovine serum (FBS) were obtained from GE Healthcare Life Sciences (Buckinghamshire, Germany). Ninety-six-well microtiter immunoplates were from Nunc (Roskilde, Denmark). Recombinant human TNFα was obtained from Biosource (CA, USA). 2.5S NGF (from mouse submaxillary glands) was from Promega (MA, USA). l-glutamine, antibiotics (penicillin/streptomycin mixture), β-mercaptoethanol, bovine serum albumin (BSA), 3-(4,5-dimethylthiazol-2-yl)-2-5-diphenyltetrazolium bromide (MTT), and Tween 20 were purchased from Sigma (MO, USA). Rabbit anti-NGF polyclonal antibody and molecular weight marker (10–250 kDa) were obtained from Pierce (Rockford, USA). Rabbit anti-BDNF polyclonal antibody was from Bioss (MA, USA). Alkaline phosphatase-conjugated anti-rabbit/anti-mouse IgG antibody was from Cell Signaling (MA, USA). 5-bromo-4-chloro-3-indolyl phosphate disodium salt (BCIP) and nitro-blue tetrazolium (NBT) were from Carl Roth (Karlsruhe, Germany). NucleoSpin RNA isolation kit was obtained from Macherey-Nagel (Düren, Germany). Methanol, glycine, and sodium phosphate were bought from POCH (Katowice, Poland).

The proline-rich polypeptide complex (PRP) was prepared from ovine colostrum according to the procedure of Janusz et al. (1981).

The nonapeptide fragment of PRP (NP) Val-Glu-Ser-Tyr-Val-Pro-Leu-Phe-Pro was obtained by chemical synthesis at Lipopharm (Gdańsk, Poland).

Human astrocytoma cell line U87 was obtained from American Type Culture Collection (ATCC, USA).

### Cell culture

**Fig. 1:**
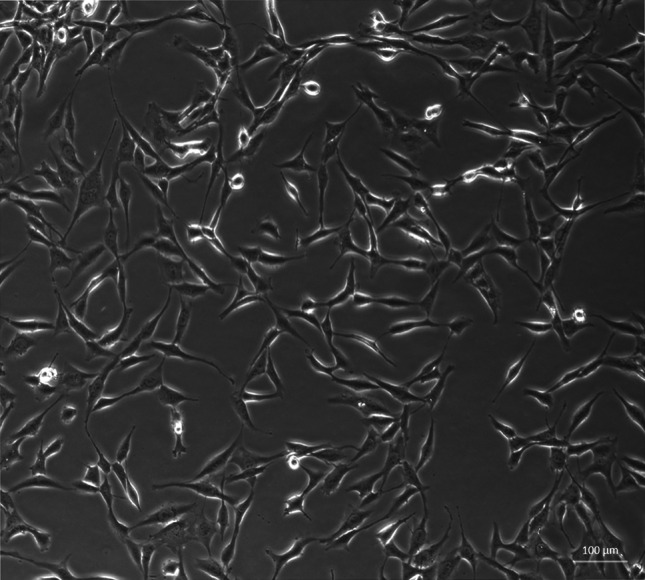
Human astrocytoma cell line U87 (Axiovert, Zaiss, ×10)

U87 astrocytoma cell line (Fig. 1) was maintained in 75 cm^2^ tasks under 5 % CO_2_/95 % humidified air at 37 °C in DMEM, supplemented with 10 % FBS, antibiotics (penicillin and streptomycin mixture), and 2 mM l-glutamine. U87 cells were grown to confluence and the medium was changed every 3 days. When cells were 90 % confluent, the cells were detached by trypsin + EDTA mixture (3 min, 37 °C), washed 3× with Dulbecco culture medium, and counted. Only the cells from passages 2–6 were used in experiments.

### Cell Viability Assay

Cell viability was measured by quantitative colorimetric assay with MTT (Denizot and Lang 1986). U87 cells were seeded in 96-well plates (1 × 10^4^/well) and incubated for 24 h with inducers: TNFα (50, 500, and 1000 pg/ml) or PRP/NP (0,1 and 10 μg/ml). Cells maintained without inducers were used as negative control. Formazan products were quantified by measuring absorbance at 570 nm with Microplate Reader (Enspire 2300 Multilabel Reader, Perkin Elmer, MA, USA). All the experiments were repeated three times. The cell viability was expressed as the percentage of living cells incubated with inducers *vs* control.

### Stimulation of U87 Cells

U87 cells (1 × 10^6^/ml for NGF/BDNF induction and 3 × 10^5^/ml for IL-6 induction) were suspended in serum-free DMEM medium and plated in 60-mm culture dishes. PRP (0.1 and 10 μg/ml), NP (0.1 and 10 µg/ml), or TNFα (50 pg/ml) used as a reference sample were applied to cells and incubated at 37 °C, 5 % CO_2_ for 3 h to induce NGF expression and 24 h to induce NGF, BDNF, and IL-6 production. NGF and BDNF produced by U87 cells were measured in supernatants collected and next concentrated by Ultra centrifugal filters (Ultracel-3 kDa, Amicon) to a volume of 100 μl.

### Measurement of NGF, BDNF, and IL-6 by ELISA

NGF, BDNF, and IL-6 secreted from cultured U87 cells in the presence of peptides were determined using the NGF Emax ImmunoAssay System (Promega, MA, USA), BDNF Emax ImmunoAssay System (Promega, MA, USA), and BD Opt EIA Human IL-6 ELISA Set (BD Pharmingen, CA, USA) according to the procedures provided by the manufacturer.

### Western Blotting

U87 cells (5 × 10^5^/ml) were seeded onto 60-mm culture dishes in Dulbecco culture medium and cultured for 24 h at 37 °C. After this time, the medium was replaced with Dulbecco without serum, and PRP (0.1 and 10 μg/ml), NP (0.1 and 10 µg/ml), or TNFα (50 pg/ml) were added and incubated for 24 h for induction of NGF/BDNF production. Next, the culture media from control and peptide-treated cells were collected and concentrated by Ultra centrifugal filters (Ultracel-3 kDa, Amicon) to a volume of 100 μl. The protein concentration was determined by the bicinchoninic acid assay. Samples of 50 μg protein in loading buffer were boiled for 5 min and loaded on SDS 12 %-polyacrylamide gel electrophoresis. Then the gels were transferred to a nitrocellulose membrane (0.22 μm, Protran, Sigma). Blots were blocked for 1 h at room temperature with 5 % non-fat dried milk in Tris-buffered saline (10 mM Tris–HCl pH 8.0,150 mM NaCl, 0.05 % Tween 20). The blots were then probed overnight at 4 °C with polyclonal rabbit anti-βNGF antibodies (1:1000) or polyclonal rabbit anti-BDNF antibodies (1:1500) washed and then incubated with secondary, alkaline phosphatase-conjugated anti-rabbit/anti-mouse IgG antibody (1:10,000) for 1 h at room temperature. Immunocomplexes were visualized using a NBT/BCIP substrate and analyzed in Molecular Imager ChemiDoc MP Imaging System with Image Lab 5.2 Software (Bio-Rad).

### Real-Time Quantitative PCR

Total RNA was extracted from U87 cells, peptide-stimulated or non-stimulated cells after 3 h of incubation using a NucleoSpin RNA isolation kit (Macherey-Nagel) following the manufacturer’s protocol. Total cDNA was used as starting material for real-time quantitative PCR with GoTaq qPCR Master Mix with BRYT Green dye (Promega) on a real-time PCR system (CFX Connect Real-time System, Bio-Rad). For amplification of specific genes, the following primers were used: *NGFβ*, forward 5′-GGGAGCGCAGCGAGTTTTG-3′, and reverse 5′-TTAAACAGCCTGGGGTCCAC-3′. For mRNA quantification, the housekeeping gene hypoxanthine phosphoribosyltransferase 1 (*HPRT*) was used as a reference point using the following primers: *HPRT* forward 5′-AGCTTGCTGGTGAAAAGGAC-3′, and reverse 5′-TTATAGTCAAGGGCATATCC-3′. Real-time PCR data were analyzed using the 2^−ΔΔ*C*t^ method.

### Statistical Analysis

Statistical analyses were performed using the software package Statistica 6 by StatSoft. Results are presented as median ± quartiles (25–75 %) and min–max. Statistical significance of differences between the values of analyzed samples was evaluated by the nonparametric Wilcoxon test for NGF determination and Student’s *t* test for MTT, NGF expression, BDNF, and IL-6 determination. A value of *p ≤ 0.05 was considered statistically significant.

## Results

### The Effect of PRP and NP on U87 Cell Viability

**Fig. 2:**
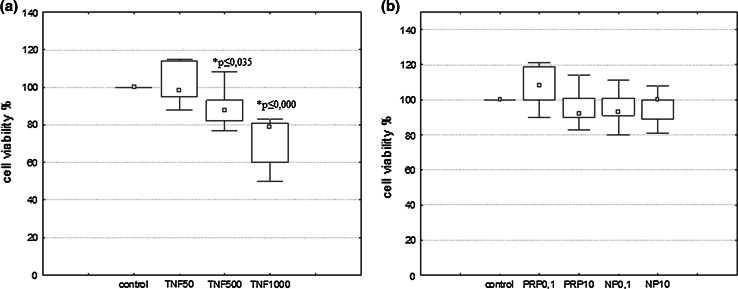
Effect of TNF (**a**), PRP and NP (**b**) on viability of U87 human astrocytoma cell line. U87 cells (1 × 10^4^/well) were exposed to PRP, NP (0.1 and 10 μg/ml), or TNFα (50, 500, and 1000 pg/ml) for 24 h. Cell viability was evaluated using MTT assay. The data are median ± quartile (25–75 %) and min–max (*n* = 5–6). *p ≤ 0.05 statistically significant difference in sample value versus control

Cell viability of U87 cells treated with the indicated drugs was evaluated by MTT assay. TNFα used at dose 50 pg/ml showed no toxic effect on U87; however doses 500 and 1000 pg/ml induced 11 and 26 % reduction of viability, respectively (Fig. 2a). The results also revealed that PRP and NP at doses 0.1 and 10 μg/ml are not cytotoxic to U87 cells (Fig. 2b) similar to 50 pg/ml of TNFα checked as a reference sample.

### The Effect of PRP and NP on NGF and BDNF Protein Level

**Fig. 3:**
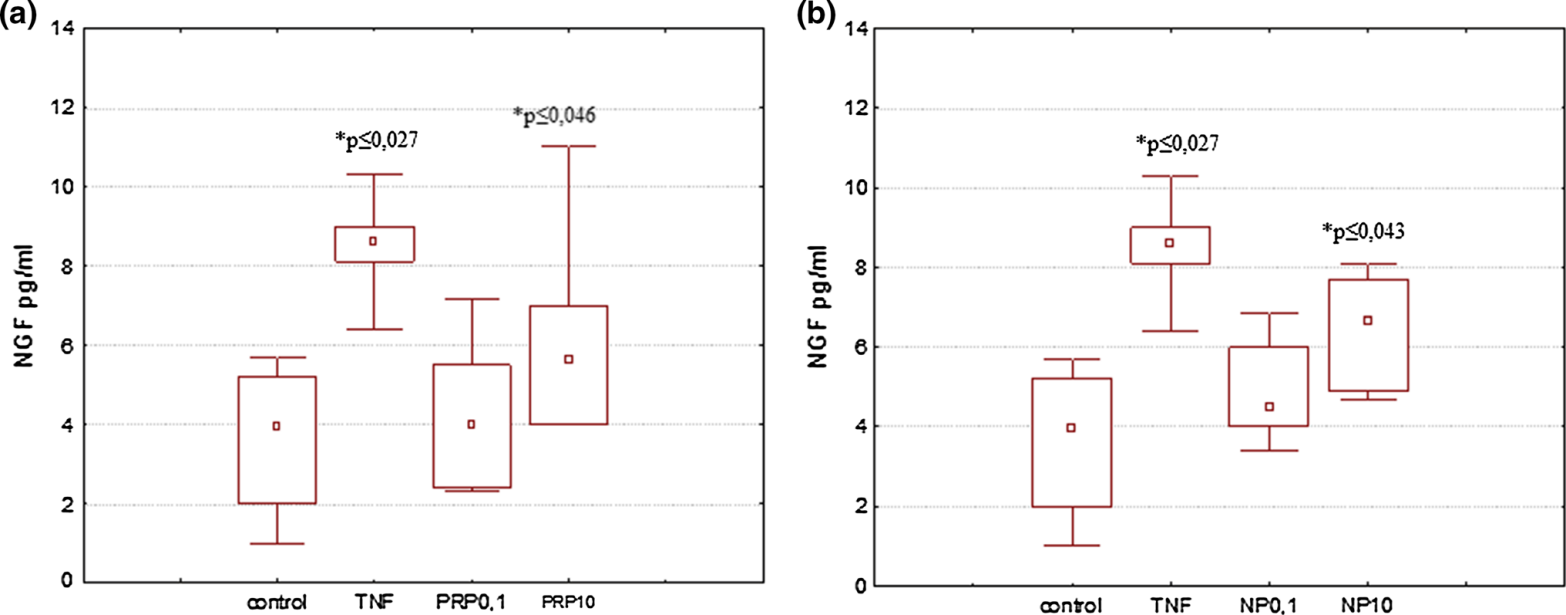
NGF induction in human astrocytoma cell line U87 by PRP (**a**) and NP (**b**). U87 cells (1 × 10^6^/ml) were incubated with TNFα (50 pg/ml) used as reference sample, PRP (0.1 and 10 μg/ml), or NP (0.1 and 10 μg/ml) for 24 h. Supernatants were harvested, concentrated, and then analyzed for NGF protein level by ELISA as described in Materials and Methods. Results are presented as median ± quartile (25–75 %) and min–max (*n* = 5–6). *p ≤ 0.05 statistically significant difference in sample value versus control

**Fig. 4:**
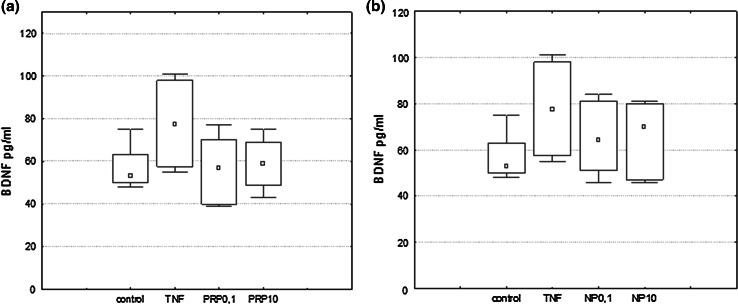
BDNF induction in human astrocytoma cell line U87 by PRP (**a**) and NP (**b**). U87 cells (1 × 10^6^/ml) were incubated with TNFα (50 pg/ml) used as reference sample, PRP (0.1 and 10 μg/ml), or NP (0.1 and 10 μg/ml) for 24 h. Supernatants were harvested, concentrated, and then analyzed for BDNF protein level by ELISA as described in Materials and Methods. Results are presented as median ± quartile (25–75 %) and min–max (*n* = 5). *p ≤ 0.05 statistically significant difference in sample value vs control

The effect of the polypeptide complex PRP and nonapeptide NP on total extracellular NGF and BDNF levels (pro-forms and mature form) was determined by ELISA assay. It was shown that both PRP (Fig. 3a) and NP (Fig. 3b) at a dose of 10 μg/ml, as well as TNFα (50 pg/ml) used as a reference sample (Fig. 3b) stimulate human astrocytoma cell line U87 to release significant amounts of NGF. Extracellular NGF level increased significantly from 4.16 pg/ml in the control sample to 7.6 pg/ml and to 6.8 pg/ml in the presence of PRP and NP, respectively. Astrocytes are able to secrete constitutive amounts of BDNF, but no effect of PRP (Fig. 4a) and NP (Fig. 4b) on BDNF release was observed.

**Fig. 5:**
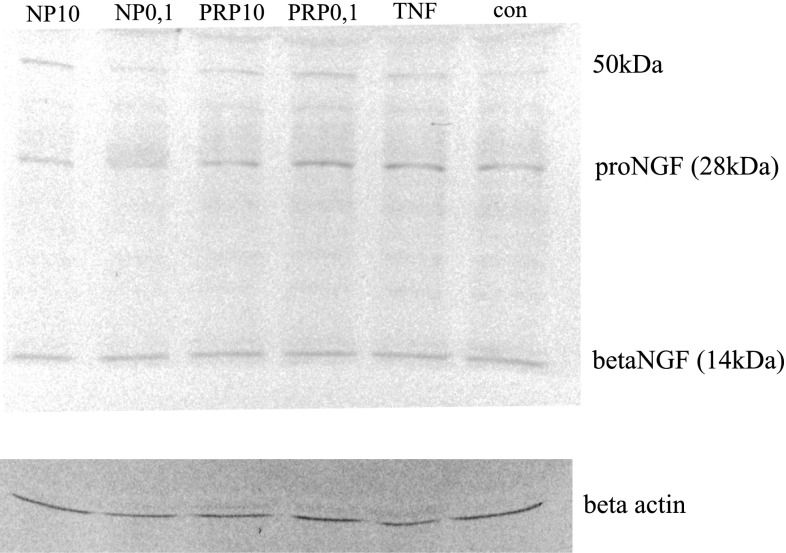
Western blotting analysis of NGF forms secreted by astrocytoma cell line U87. Supernatants from control and compound-treated cells were separated in SDS-PAGE, then transferred to nitrocellulose membrane. NGF forms were detected with the use of specific polyclonal antibodies anti-βNGF. Immunocomplexes were visualized using a NBT/BCIP substrate and analyzed densitometrically in Molecular Imager ChemiDoc MP Imaging System with Image Lab Softwere (BioRad). The representative immunoblot is presented

Results obtained from ELISA demonstrated that the concentration of the total NGF released by PRP/NP-treated U87 astrocytoma cells was higher than in control cells. The molecular forms of NGF and BDNF released were additionally identified by Western blot. As shown in Fig. 5, the 28-kDa pro-form and 50-kDa protein probably glycosylated proNGF and mature NGF which migrates at close to 14 kDa were detected. The intensities of bands related to proNGF and mature NGF bands are comparable to the control sample. The relative ratio of mature NGF to proNGF in particular samples was also determined. No statistically significant differences in the ratio of both isoforms per sample were observed. It was also revealed that BDNF released by U87 cells exists only as a pro-peptides, without mature form (data not shown).

### The Effect of PRP and NP on NGF mRNA Expression

**Fig. 6:**
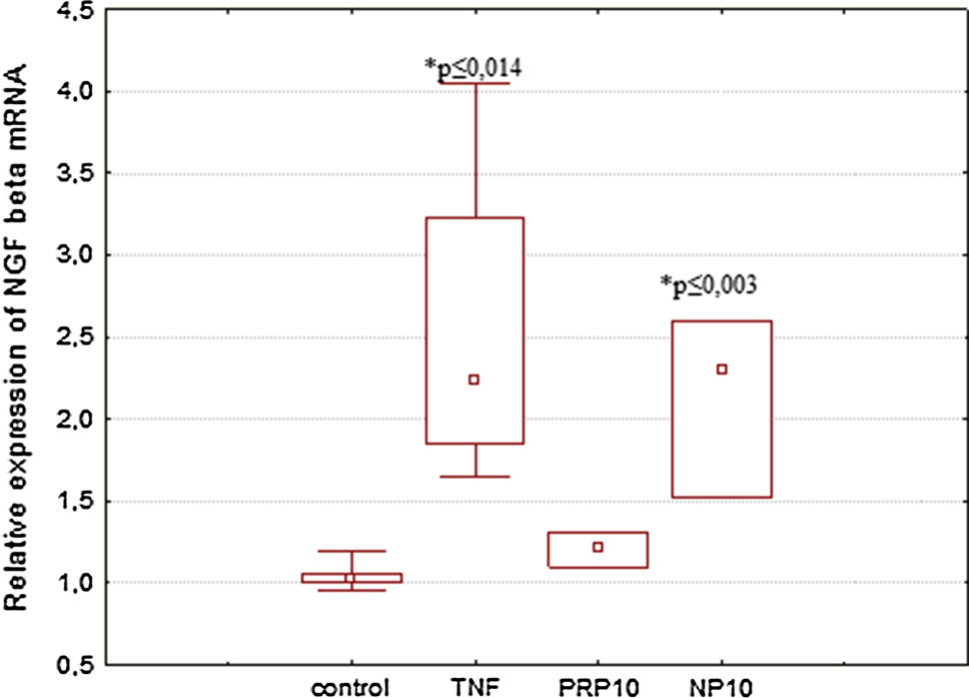
Expression of NGF mRNA in human astrocytoma cell line U87 in response to PRP and NP. U87 cells (1 × 10^6^/ml) were incubated with TNFα (50 pg/ml), PRP (10 μg/ml), or NP (10 μg/ml). After 3 h of stimulation, expression of NGF mRNA was measured by real-time PCR. Results are presented as median ± quartile (25–75 %) and min–max (*n* = 3–5). *p ≤ 0.05. Statistically significant difference in value versus control

It was observed that the NGFβ mRNA expression was significantly increased in U87 cells incubated with NP at a dose of 10 μg/ml (Fig. 6). The significantly high level of NGF mRNA, 2× control value, was checked after 3 h of treatment and decreased after 24 h to the control level. This result is comparable to the effect of TNFα used as a reference sample. The results obtained correlate positively with the increased production of NGF detected by ELISA.

### The Effect of PRP and NP on IL-6 Production

**Fig. 7:**
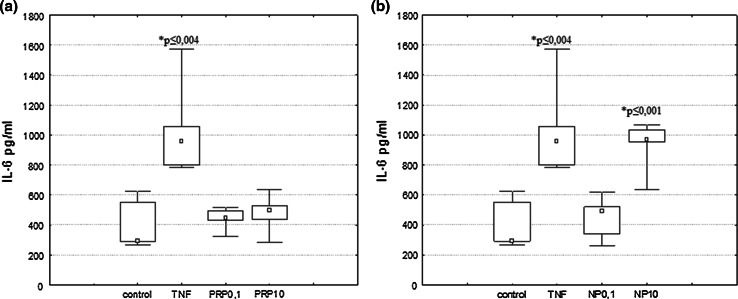
IL-6 secretion by human astrocytoma cell line U87 after PRP (**a**) and NP (**b**) treatment. U87 cells (3 × 10^5^/ml) were cultured in the presence of PRP (0.1 and 10 μg/ml) or NP (0.1 and 10 μg/ml) for 24 h. TNFα (50 pg/ml) was used as reference sample. Supernatants were harvested and analyzed for IL-6 level by ELISA as described in Materials and Methods. Results are presented as median ± quartile (25–75 %) and min–max (*n* = 5). *p ≤ 0.05. Statistically significant difference in value versus control

Control human astrocytoma cell line U87 secretes interleukin-6 at the level 386 pg/ml. After 24 h of incubation with 50 pg/ml of TNFα used in our experimental conditions as a reference sample, the IL-6 level in supernatants increased to 259 % of the level of untreated cells (1000 pg/ml) (Fig. 7a, b). To evaluate the IL-6-inducing activity of PRP and NP in human astrocytoma U87 cells, the polypeptide complex PRP and its nonapeptide fragment were added to the cells at doses of 0.1 or 10 μg/ml. It was observed that under our experimental conditions, PRP did not induce secretion of IL-6 (Fig. 7a). However, the level of IL-6 released after 24 h of incubation with 10 μg/ml of NP significantly increased to 891 pg/ml (Fig. 7b).

## Discussion

Astrocytes are involved in regulation of the brain microenvironment, controlling ion homeostasis, synaptic transmission, and neuronal excitability. Astrocytes can produce and release to the extracellular matrix a wide spectrum of biologically active substances responsible for regulation and control of neuron function (Barkho et al. 2006; Oh et al. 2009; Sofroniew and Vinters 2010; Pirttinaki and Parri 2013) Among them are neurotrophins represented by NGF, BDNF, and NT 3, 4/5, and 6 (Lessmann et al. 2003; Twiss et al. 2006; Reichardt 2006; Bartkowska et al. 2010; Aloe et al. 2012). Neurotrophins play an essential control role in the developmental neurogenesis, and also are important for adult neurogenesis, survival of neurons, and its protection against damaging signals (for references see Markiewicz and Lukomska 2006, Miklič et al. 2004). Astrocytes play an important role under physiological conditions, and also can protect neurons against damage during brain injury or in neurodegenerative processes accompanying Alzheimer’s disease or Parkinson’s disease (Lessmann et al. 2003; Allen et al. 2013; Angelowa and Abramow 2014).

The use of primary human astrocytes from central nervous system tissue is extremely difficult and is limited by its high sensitivity and limited viability. Therefore, transformed astrocyte-like cell lines can be used alternatively for in vitro examination. Immortalized cell lines with astrocyte-like morphology such as U87, U373, or 1321N1 originally derived from human malignant tumors constitute relatively good model system for astrocytes in vitro because of their functional similarity to normal astrocytes (Wagoner et al. 1999; Nishiguchi et al. 2003; Wong et al. 2010; Yoshida et al. 2011; Saeed et al. 2015). During previous studies on the proline-rich polypeptide complex (PRP), activities were shown that both PRP complex and its nonapeptide fragment NP at low doses ranging from 0.01 to 10 μg/ml increase neuronal survival stimulate neuronal proliferation and induce neuritogenesis of PC12 cells (Basci et al. 2005; Schuster et al. 2005; Bourhim et al. 2007; Douraghi-Zadeh et al. 2009; Zabłocka et al. 2014). Based on published results obtained on human astrocytoma cell lines used to examine the molecular signaling and mechanisms connected with protection of neuronal cells (Wagoner et al. 1999; Deb et al. 1999; Nischiguchi et all., 1999; Mori et al. 2008; Wang et al. 2010a, b; Yoshida et al. 2011; Li et al. 2014; Saeed et al. 2015), the purpose of the present study was to investigate the ability of proline-rich polypeptide complex PRP and its nonapeptide fragment NP to up-regulate the secretion of neurotrophins on the model of human astrocytoma cell line U87.

Our present study demonstrated that human astrocytoma cells U87 can produce and release to the extracellular space constitutive amounts of NGF and BDNF protein in accordance with the observations of van Wagoner et al. (1999), Mori et al. (2008), Angelucci et al. (2011) and Mele and Jurič (2014). We showed for the first time that both the PRP complex (Fig. 3a) and the nonapeptide fragment of PRP (Fig. 3b), at a dose of 10 μg/ml, possess NGF-inducing activity. No significant effect of PRP (Fig. 4a) and NP (Fig. 4b) on the extracellular level of BDNF was observed. To explain the cellular mechanisms of NGF production by U87 cells, the effect of PRP and NP on NGF expression was evaluated using real-time PCR. The increased level of NGF mRNA was observed only in NP-treated cells (Fig. 6). Because nonapeptide NP is one component of the PRP complex, we can speculate that the content of nonapeptide in 10 μg/ml of the whole PRP complex is too low to activate NGF expression, and higher doses than 10 μg/ml of PRP should be used to obtain an effect comparable to 10 μg/ml of isolated nonapeptide NP.

NGF synthesis in primary astrocytes is connected with ERK 1/2 MAP kinase activation as was shown by Xu et al. (2013) and Park et al. (2006). In our previous work, it was shown that PRP is able to activate ERK 1/2 MAP kinases (Zabłocka et al. 2014). We can suppose that an increased level of βNGF may be also connected with the ability of the PRP complex to activate the ERK1/2 kinase signaling pathway or with the effect of PRP on NGF release to the extracellular space (Lessmann et al. 2003; Reichardt 2006).

It was observed by Kuno et al. (2006) that exogenous TNFα and also TNFα produced by astrocytes can induce NGF production by astrocytes. It was shown that the astrocytoma cell line U87 expressed and released increased amounts of NGF in response to 50 pg/ml of exogenous TNFα. It was previously reported by us that both PRP and NP are good inducers of TNFα secretion in whole human blood cells and human peripheral blood mononuclear cells (Zabłocka et al. 2001, 2007). It is possible that PRP/NP-treated U87 cells can secrete TNFα, which acts in an autocrine manner and contributes to NGF production.

It was shown that neurotrophins are synthesized intracellularly as pro-peptides, can be secreted, and cleaved extracellularly to their active forms by serine protease plasmin and by selective metaloproteinases (Lee et al. 2001; Twiss et al. 2006; Allard et al. 2012). Continuous cleavage of pro-NGF and pro-BDNF by proteases generates intermediate forms and the final, mature 13 kDa NGF and 14 kDa BDNF. ELISA assay detects both mature forms NGF and pro-NGF. With the use of Western blotting, in the supernatants obtained from PRP- and NP-treated U87 cells, a 14 kDa signal corresponding to the mature NGF, 26 kDa corresponding to the pro-NGF, and additionally to the 50 kDa form, probably high glycosylated NGF, was identified (Fig. 5). In the case of BDNF, only the pro-peptides, without the mature BDNF form, were identified (data not shown).

The biological role of pro-NGF is still under investigation, and it is rather controversial. Lee et al. (2001) and (Wang et al. 2010a, b) reported that pro-NGF promotes apoptosis of neurons. In contrast, Fahnestock et al. (2004) found that pro-NGF neurotrophic activity is similar to mature 2.5S NGF but with fivefold lower activity. They proposed the hypothesis that pro-NGF may be responsible for the neurotrophic activity in physiological conditions in most tissues, but injury can increase the proteolytic processing of pro-NGF to mature NGF with about five times higher neurotrophic activity that of pro-peptide. In turn, Masoudi et al. (2009) proposed a mechanism in which neurotrophin or apoptotic activity of pro-forms of neurotrophins is regulated by the relative level of NGF receptors expressed by neuronal cells.

Despite of evidences suggesting the pathological role of interleukin-6 (IL-6) in neurodegeneration processes, little is known about the biological activity of IL-6 in the CNS, and its role is still controversial (for references see Barkho et al. 2006). It has been shown that interleukin-6 derived from astrocytes play an important role in such functions as coordination of neuro-immune responses, protection of neurons from insult and also neuronal growth, survival, and differentiation (Drapeau et al. 2003; Barkho et al. 2006; McAfoose and Baune 2009; Oh et al. 2010) Also, in normal brain, IL-6 plays an important role in memory formation (Hryniewicz et al. 2007). On the other hand, Balschun et al. (2004) found that overexpression of IL-6 in the CNS in pathological conditions affects memory and learning processes. There is some increasing evidence for neuroprotective effect of IL-6—NGF interaction in the brain. Sterneck et al. (1996) proposed that IL-6 may act as an enhancer of NGF signaling by increasing ERK1 MAP kinase activity and induction of expression of target genes controlling the survival/differentiation of neurons. Synergistic activity of IL-6 and NGF which leads to induction of neurite extension was also observed by Suzuki et al. (1998). Neuritogenic activity of astrocyte-released interleukin-6 may also be connected with the ability of low doses of this cytokine to stimulate BDNF synthesis in neurons (Bartkowska et al. 2010).

In our previous studies, we found that PRP complex and nonapeptide NP can modulate IL-6 release from peripheral blood mononuclear cells and human whole blood cells (Zabłocka et al. 2001, 2007). In the case of U87 cells, we observed that only nonapeptide NP stimulate cells to release significant amounts of IL-6 (Fig. 7b).

Our results obtained with the human astrocytoma cell line U87 show the effective induction of NGF and IL-6 by PRP/NP. This suggests the possibility of their use as neurotrophin inducers. Improving both NGF and IL-6 secretion can be important during neonatal development, in control of “adult” neurogenesis in the hippocampus and neocortex and also in the pathogenesis of diseases such as Alzheimer’s disease, Parkinson’s disease, or depression (Bruno et al. 2009; Angelucci et al. 2011). Taking into account that the NP fragment, but not the whole PRP complex, induces neurotrophic action of IL-6, it is possible to propose particular components of the full PRP complex with selective activity as therapeutic agents in selected disorders.

NGF-dependent pathway plays a very important role in neonatal development, aging processes, and in the etiopathogenesis of neurodegenerative diseases. A better understanding of the NGF-dependent signaling in aging processes is very important for planning the therapeutic and/or preventive strategy. Difficulties connected with the use of recombinant NGF in the therapy of neurodegenerative diseases prompted the search for new therapeutic strategies (Allen et al. 2013). Naturally occurring substances such as proteins and peptides possessing regulatory properties seem to be very promising. One of them is the colostrum-derived proline-rich polypeptide complex PRP and alternatively its nonapeptide fragment NP, stable and easy to obtain in chip chemical synthesis. The positive effects of PRP on survival, neuroprotection, and neuritogenesis of PC12 cells were shown. Its role in the amplification of signals controlling the survival and differentiation of neurons is also observed when there is a deficit of NGF(for references see Janusz and Zabłocka 2010). Results obtained show that PRP/NP is able to increase the level of neurotrophic substances released by human astrocytoma cell line U87.

## Conclusions

In conclusion, our observations have shown that the proline-rich polypeptide complex PRP and its nonapeptide fragment NP up-regulate neurotrophic activity of astrocytoma cell line U87 by increase of NGF synthesis and its release into the extracellular space. It makes NGF more available to neurons and in turn may support survival and function of neuronal cells. Also, nonapeptide-dependent increased production of IL-6 can enhance the neurotrophic signaling of NGF in the CNS.
